# Progress in Surgery

**Published:** 1900-04-07

**Authors:** 


### Progress in Surgery.

### SURGERY OF THE VERMIFORM APPENDIX.

Keetley[1] suggests the following method of operating, which, though not always indicated, is safe whether on the the first or last day of an acute, subacute, or chronic attack. It takes into consideration the path by which the pus, when pus forms, usually mounts to the inguinal region of the abdominal wall, and it goes to meet it. At the same time it keeps as often as possible outside the peritoneal cavity. The surgeon proceeds somewhat as if he wished to tie, extra-peritoneally, the common iliac artery. (1) The skin is incised above the anterior superior iliac spine and the outer half of Poupart's ligament in a line parallel to the latter. (2) the external oblique aponeurosis is divided to the extent of the skin incision; (3) the deeper muscles are penetrated as much as possible by separation of their fibres and towards the outer part of the skin incision by cutting with scissors; (4) the transversalis fascia is divided; (5) the peritoneum is carefully separated from the iliac fascia, the surgeon at the same time palpating for the deep surface of the appendix, or for the inflammatory thickening around it; (6) a strong light is thrown into the depths of the wound, and signs of inflammatory infiltration of the tissues such as indicate the neighbourhood of pus are looked for; (7) if pus is found the infiltrated tissues are carefully torn open with two pairs of dissecting forceps—not cut or roughly punctured; and (8) whether pus is found or not two drainage tubes of medium calibre are carried to the bottom of the wound. If a collection of pus is swabbed out the greatest gentleness should be employed. It is better not to use the douche or syringe. If the appendix is in the centre of adhesions, possibly bounding pus, and the thickened mass floats comparatively free in the abdominal cavity, then (1) if it can be done without tension, bring the inflamed mass and fix it by suture through the healthy tissues to some part of the parietal wound, and leave that part of the wound open; (2) if the above course cannot be followed without dragging on the bowel either carry a drain down to the inflammatory mass or leave it alone altogether.

Hernia after operation for appendicitis is a danger dwelt upon by Woolsey,[2] who emphasises the following facts: (1) In operations for inflamed appendix when pus is present the first consideration is the life of the patient, and the second the avoidance of disagreeable sequelæ, of which ventral hernia is the most common, and hence the most serious. (2) Post-operative ventral hernia may be largely avoided in spite of the necessary use of drainage. McBurney's inter-muscular or muscle-splitting incision should be practised; it avoids the division of nerves beyond obviating mechanical and cicatricial weakening of the wound. The wound, again, in order to avoid hernia, must be closed as much as possible by sutures. Provisional or secondary sutures are then passed into the part left open for drainage. On the other hand, instead of these provisional sutures, the gauze-drain may be removed early, so as to allow the walls of the cavity occupied by the gauze to fall together before they have become so firm and rigid as to remain apart, leaving the cavity to fill up by granulation. After pushing the gauze through the nearly closed wound, a piece of indiarubber tissue is wrapped round the portion passing through the wound. This prevents the gauze from sticking to the edge of the wound, and thus allows it to be removed on the second day without any dangerous traction in structures beginning to heal. Kelly[3] says that no one factor can be held answerable for the production of all cases of appendicitis. Although the disease is almost without exception the consequence of micro organismal infection, it is rather of complex pathogenesis, and no one morbific agent is provocative of all attacks. It is because of the anatomical and physiological peculiarities of the appendix that factors innocuous in the intestine, or morbific agents capable of being successfully counteracted by the physiological activities of the intestine, become in the appendix of heightened virulence, and engender the most disastrous consequences. The factors that in the appendix give rise to increase in the virulence of bacteria, normally present in the intestine, are such as interfere with thorough drainage of the organ. Edebohls,[4] in a most interesting review of the history and literature of appendicitis, says that the gridiron incision of McBurney represents the most modern thought and fully satisfactory technics in regard to the incision of the anterior abdominal wall, practised anywhere between the outer borders of the recti and the erector spinæ muscles on either side; not only for appendicitis, either acute or chronic, but also for other intra-abdominal conditions exceptionally best approached within the limits stated. Edebohls originated inversion of the entire, uncut appendix, the only procedure which does away with the necessity of opening the bowel, and with the resultant risk of infection. Wyeth[5] advocates operation in two stages if there has been a considerable exudate, or if pus has formed. When an abscess has formed, and this abscess can be opened directly through any part of the abdominal wall in which adhesions exist which enable the operator to drain the septic contents off without entering the general peritoneal cavity, Wyeth maintains that it is better practice to do this than to attempt removal of the infectious mass. The vast majority of these patients recover, and only a very small proportion of them have a second attack. Should a second attack occur, he would carry out the same line of practice, and insist upon a removal of the diseased appendix after the acute infection had passed, and in what is called the quiescent period. Barling[6] reports 117 cases operated upon for appendicitis. In 42 the operation was performed in a quiet interval, and only one death occurred. The remaining 75 cases he divides into four groups: (1) The "safe" abscess, with the pus so localised by adhesions that the surgeon opens into it without danger of infecting the general belly cavity; (2) "non-adherent abscess," in which the pus is shut off from the general peritoneal cavity, but the operator has to open the general cavity to seek it; (3) "subacute," widespread suppurative peritonitis; (4) "acute fulminating peritonitis." Group 1 comprised 19 cases, with 1 death; group 2, 22 cases, no deaths; group 3, 9 cases, with 3 deaths; group 4, 26 cases, with 11 deaths. The total mortality is 20 per cent., which the author considers in most instances to be the mortality of delay. The great thing in recognising a case suitable for operation is not to take any one point as essential to diagnosis, and to dwell not so much upon the absence of any one particular feature as upon the intensity of such symptoms as are present. Mitchell[7] records that of 1,400 cases of appendicitis collected from various sources in the last ten years, 7 per cent. had true foreign bodies, and not including fæcal concretions, which were present in 315 cases. The most common foreign bodies are gall stones, round worms, splinters of bone, bristles and pins. Mitchell collected 28 cases of pins found in the appendix, 19 being males, and nine females. The resulting appendicitis was of a very variable type, but in the majority rapid perforation and abscess formation followed the initial symptoms. Light foreign bodies, like grape seeds and cherry stones, are in reality exceptional, their frequency having been overestimated to lack of careful discrimination from fæcal concretions, which are the most commonly occurring bodies in this situation. Moullin[8] maintains that if a case of appendicitis does not show definite signs of improvement within 36 hours, it is wiser to operate than to trust to chance. The limit of 36 hours is chosen because it is unusual for either suppuration or gangrene to have spread very widely within that time, because, if the symptoms definitely begin to subside by then, there is a reasonable hope that the case will terminate favourably; and because if the effusion continues to increase after that time no one can say what may happen. Even if suppuration does not occur, it is very unlikely that resolution will ever be complete, or that the whole of the exudation will be absorbed. Some of it is almost certain to become organised, and to tie the appendix down and distort it until complete recovery is impossible. In judging of the advisability of operation negative evidence is valueless. One well-marked symptom is enough, and not only justifies but compels operation. If the onset is sudden, resembling that of perforative peritonitis, particularly if it is followed by severe pain or meteorism; if there is evidence of septic poisoning; if the pulse continues to be rapid, particularly if it increases in rapidity after 36 hours, whether the temperature is high or low; if the pain continues to be severe; if there is increasing abdominal distension, with tenderness; if there is not a distinct improvement at the end of 36 hours; or if at any time in the course of a mild attack the symptoms suddenly become grave, operation is regarded as imperative. As opposed to this Berry[9] says that every acute case of appendicitis should be treated upon its merits, and not in accordance with hard and fast rules, that the early operation for acute appendicitis does not, in the hands of most surgeons, yield very satisfactory results unless performed upon the slighter cases only, and that a delay of a few days will in the great majority of cases allow of the performance of a smaller, safer, and, on the whole, more satisfactory operation. Tixier[10] operated on an almost moribund patient with general suppurative peritonitis arising from appendicitis. He carefully placed strands of gauze drain in every direction, in Douglas's pouch, under the liver, and also under the spleen, and found no visceral or parietal adhesion. Thus acute general purulent peritonitis of appendicular origin need not make the surgeon despair.

[1] Lancet, Jan. 13, 1900, p. 87. [2] Brit. Med. Jour., 1899, Vol. II.; Epitome, No. 244; and Med. Rec., April 1, 1899. [3] Columbus Med. Jour., Dec., 1899, p. 523. [4] Med. Rec., Nov. 25, 1899, p. 774. [5] Med. Rec., Jan. 6, 1900, p. 44. [6] Edin. Med. Jour., Dec., 1899; and Med. Rec., Jan. 6, 1900, p. 33. [7] Brit. Med. Jour., Sept. 9, 1899; Epitome, No. 193; and Johns Hopkins Bull., Jan., Feb., and Mar., 1899. [8] Clin. Jour., Dec. 20, 1899, p. 139. [9] Lancet, Dec. 30, 1899, p. 1,858. [10] Brit. Med. Jour., Dec. 30, 1899; Epitome, No. 498.

